# Impact of Raw Material Particle Size on Processing, Physical Quality and In Vivo Performance of Grain Sorghum and Wheat-Based Extruded Feed for Tilapia *Oreochromis niloticus*

**DOI:** 10.3390/ani16060858

**Published:** 2026-03-10

**Authors:** Tucker Graff, Donald A. Davis, Sajid Alavi

**Affiliations:** 1 Department of Grain and Food Science, Kansas State University, Manhattan, KS 66506, USA; tuckerg@ksu.edu; 2 School of Fisheries, Aquaculture and Aquatic Sciences, Auburn University, Auburn, AL 36849, USA; davisda@auburn.edu

**Keywords:** aquaculture, extrusion, sorghum, particle size, feed processing

## Abstract

This research compared grain sorghum with wheat as an ingredient in extruded, floating tilapia feed, and also studied the impact of pre-extrusion grinding intensity or hammer mill sieve size on extrusion parameters, final product quality and animal performance. With an increase in grind size of the diets from 0.61 to 1.27 mm, higher specific thermal energy was observed; however, specific mechanical energy decreased, leading to lower expansion (pooled bulk density of 405.6 g/L versus 441.5 g/L). Grain source also impacted pellet expansion and quality, with sorghum-based aquatic feed pellets having higher piece density than wheat-based pellets (pooled average of 0.52 g/cm^3^ versus 0.48 g/cm^3^) and lower water absorption (pooled average of 255.7% versus 334.4%). Digestibility trends with respect to grain and grind size were not consistent for Nile tilapia fed different extruded diets, but results from a 12-week growth trial showed that tilapia fed the sorghum-based diet had a higher weight gain as compared to wheat-based diets (86.0% versus 81.8%). Grind size or grain did not have a statistically significant impact on feed conversion ratio (FCR), but the sorghum-based feed from medium grind had the lowest FCR of 1.03, while the FCR of other treatments ranged from 1.09 to 1.13. These results indicate that grain sorghum can successfully be incorporated into Nile tilapia diets with positive effects on both physical feed quality as well as the growth of the fish.

## Simple Summary

This study addressed the need for more sustainable and cost-effective ingredients in fish feed to support the rapidly growing aquaculture industry. The main objectives were to evaluate if grain sorghum, an affordable and drought-tolerant crop, could successfully replace grains like wheat in floating feed for Nile tilapia produced using extrusion processing, and to determine the impact of ingredient particle size on feed quality and fish growth. Results showed that sorghum-based diets resulted in significantly better growth and final weight for the tilapia compared to wheat-based diets, demonstrating that grain sorghum is a viable and promising alternative to wheat in tilapia feeds. Results also showed that grinding ingredients to an extremely fine particle size is unnecessary for good fish growth, allowing producers to save both time and money in feed manufacturing without negatively harming performance.

## 1. Introduction

Aquatic animals provide 15% of global animal protein, and 3.2 billion people rely on fish for at least 20% of their daily protein intake [1]. From 1961 to 2019, global fish consumption increased at an average annual rate of 3.1%, twice that of the average annual population growth (1.6%) [2]. This increase in consumption is higher than that of all other animal proteins, including meat and dairy products, which increased by approximately 2.1% annually [1]. More recently, world production of aquatic animals grew at an even higher average rate of 5.3% from 2001 to 2018 [1].

The global production of aquatic animals via capture fisheries and aquaculture in 2022 was 185.4 million tons. Aquaculture, defined as the rearing of aquatic animals for food purposes, contributed 130.9 million tons to this total [1]. Total aquatic production is valued at USD 406 billion, with the share of aquaculture being USD 265 billion. The increase in production in recent years has come mainly from the rapidly growing aquaculture industry, while capture fisheries have stagnated since around 1990 due to overfishing of wild fish stocks. The World Bank has forecast that aquaculture production will continue to sustain the growing global population and demand for animal protein in the coming years [3].

Compound feeds for aquaculture vary depending on target species, ingredient usage and production methods. Most aquatic feeds are produced using pellet milling or extrusion. Pellet sizes can range from under 1.5 mm in diameter for specialty ‘micro’ feeds to 2–12 mm for more traditional feeds. Aquaculture diets in the past relied heavily on fishmeal for protein requirements and for its high caloric value [4]. However, rising costs and limited supplies due to high demand and overfishing have made the replacement of fishmeal with alternative protein and energy sources vital for the long-term stability of the industry. Much of the research focus has been plant protein alternatives, but the current study is focused on cereal grains as a carbohydrate and energy source. Carbohydrates have a ‘protein-sparing’ effect in aquatic diets, as the use of a non-protein energy source to meet basic energy needs enables dietary protein to be spared and used for growth and tissue repair [5]. As the use of extrusion in the production of aquatic feed is increasing, starch content and the quality of diets is additionally important due to its role as a binding agent and impact on important pellet quality parameters such as density, durability and water stability [6]. Some of these aspects also help in preserving water quality and preventing health issues in animals. A commonly used cereal grain in aquatic feed formulations is wheat, which is not only a source of energy but also has important functional properties contributed by starch. Unfortunately, wheat is an expensive ingredient that is also used heavily in human foods. With rapid growth in aquaculture, it is natural that demand for aquatic feed is also rising quickly [7]. This has caused further volatility in the price of wheat used in aquatic feed. There is potential for cost savings and other benefits if cheaper and more sustainable sources of carbohydrates, such as grain sorghum, can be explored. With the increasing number and severity of drought conditions, as well as the declining water levels in aquifers, drought-tolerant cereal crops such as sorghum can provide a sustainable alternative. Even with the use of less water and other inputs, sorghum has been shown to have better yields per acre than other cereal crops [8]. Additionally, sorghum is a non-GMO (genetically modified organism) grain, which is important to consider when assessing its value. Globally, GMOs are divisive, facing particularly strong opposition in the European Union, and this stance has only gotten worse over the last 15 years [9].

The composition of grain sorghum varies across varieties, with data reported for starch (70.1–72%), crude protein (11.0–12.8%), total dietary fiber (8.4–10.9%), fat (3.2–3.5%) and ash content (1.5–1.9%) [10]. It has potential as a high-quality energy source in aquatic feed for a wide range of species, and its considerable starch content is ideal for good pellet quality. Research focused on the evaluation of nutritional efficacy of sorghum in aquatic animal diets has been summarized before [11]. In one study, tilapia were fed diets incorporating five different grains (wheat, corn, sorghum, rice and barley) at 25% inclusion level, with the grain sorghum-based diet showing maximum growth performance and improved protein retention efficiency over the other diets [12]. Another study on tilapia found that sorghum meal can replace up to 75% of maize (33% of the total diet) without negatively impacting growth performance or nutrient utilization [13]. Incorporation of sorghum starch up to an inclusion level of 30% in diets was found to have no adverse impact on growth performance, digestibility, body composition or enzyme activity for carbohydrate metabolism in hybrid red tilapia [14]. However, above 30% inclusion, digestibility coefficients of starch decreased significantly, while whole body composition and protein digestibility showed no differences. Research on rainbow trout and hybrid striped bass, which included three varieties of grain sorghum as a replacement for wheat flour (at 5, 10 and 20% of the diet), indicated digestible or available energy results similar to wheat flour. Reduced growth was observed in rainbow trout at higher inclusion levels when compared to hybrid striped bass, emphasizing the need for more species-specific research and formulations [15]. A key drawback of the above investigations is that processing and the physical quality of feed have been largely ignored, although these aspects can have an important role. Research conducted at Kansas State University has attempted to address these shortcomings. Sorghum was studied as a protein source in shrimp diets in the form of dried distiller’s grain (~34% protein) [16]. Two different processing methods were compared, steam pelleting and relatively high energy extrusion, with the latter leading to higher starch gelatinization and water stability of pellets. Up to 40% sorghum DDG could be used in the diets as a replacement of soybean meal without affecting shrimp growth. Extrusion is quickly becoming the primary processing technology for producing aquatic feeds. The raw material is combined with processing inputs such as water, steam, pressure and mechanical energy, with the last two setting it apart from traditional feed production methods such as pellet milling [16,17]. Energy is transferred into the extruded raw material in the form of thermal energy via steam in the preconditioner and mechanical energy in the barrel, both of which drive the extrusion cooking process [18,19,20,21,22,23]. More recently, the authors of the current study evaluated different levels of extrusion thermal energy input on the performance of wheat and sorghum-based diets fed to shrimp [24]. Sorghum was found to be a viable alternative to wheat without negatively impacting the growth performance of shrimp. However, starch gelatinization increased with preconditioning intensity and was found to be correlated with digestibility.

The focus of this investigation was the use of grain sorghum in aquatic feed for tilapia with a particular emphasis on the intensity of pre-extrusion grinding of diets on processing, pellet quality, and growth and digestibility. Particle size is an important consideration in the swine and broiler industry, having ramifications on animal health, growth rates and fecal production [25,26]. These relationships have not been investigated in aquatic species, with one study focusing on pellet size but not the size of the ground ingredients [27]. A 2020 review on ingredient strategies in the aquaculture feed industry reported that “most studies proved little indication of particle size for any of the ingredients use” [28]. Anecdotal evidence suggests that commercial producers prefer a low particle size because of the yet unsubstantiated belief that finely ground ingredients have a positive correlation with the digestibility and homogeneity of the diet. On the other hand, from an energy usage perspective, grinding to the largest possible particle size would lead to a reduction in production costs and also benefits in material handling. In addition, animal performance and costs, and raw material particle size, can also affect starch gelatinization during processing and downstream pellet quality parameters such as expansion and density. Smaller particle size has been shown to cause a greater degree of starch transformation in the extrusion of pet food diets [19,29]. This can also lead to improvements in binding and water stability in the case of aquatic feed, and also higher pellet expansion and lower bulk density. The latter can be an added benefit in the case of aquatic species that are not mandatory bottom-feeders or benthivores, where floating feed can allow better monitoring of feed consumption and minimize negative impacts on water quality.

A primary hypothesis of the study was that sorghum-based extruded feed would perform similarly to wheat-based feed in feeding trials with tilapia. It was also hypothesized that a greater degree of particle size reduction in raw diets would lead to higher expansion in the floating aquatic feed, improvements in pellet quality, and potentially also the feeding performance of tilapia. The specific goals were to evaluate the impact of hammer mill sieve size or grinding intensity of sorghum and wheat-based raw diets on the extrusion parameters, final pellet quality attributes such as water stability, and growth of Nile tilapia fed the extruded diets, and also the digestibility of the latter.

## 2. Materials and Methods

### 2.1. Experimental Design

The study constituted a 2 × 3 factorial experimental design with tilapia diets based on two grain types (wheat and red sorghum) as one independent variable, and hammermill screen sizes used in pre-extrusion grinding of the diets (0.61 mm, 1.02 mm, and 1.27 mm) as the second independent variable. This resulted in a total of 6 experimental treatments.

### 2.2. Formulations

Both wheat- and sorghum-based diets were formulated to be nutritionally complete and balanced, with approximately 36% crude protein and 8% crude fat according to the dietary requirements of Nile tilapia, as shown in Table 1. Grain sorghum was sourced from Nu-Life Market (Scott City, KS, USA), corn protein concentrate (Empyreal 75) from Cargill, Inc. (Wayzata, MN, USA) and vitamin and mineral premixes from Ziegler Bros, Inc., (Gardners, PA, USA), while all other ingredients were obtained from Fairview Mills (Seneca, KS, USA). Major ingredients were sampled and proximate analyses conducted for percent dry matter, crude protein (AOAC 990.03, nitrogen combustion method) [30], fat (AOAC 920.39, ether extract method) [30], ash (AOAC 942.05) [30] and total dietary fiber (AACC method 32-07) [31] through SDK Laboratories (Hutchinson, KS, USA). Formulas were then adjusted to account for the true nutritional value of the major ingredients to achieve similar crude protein and fat contents.

### 2.3. Grinding and Mixing

Whole grains (wheat and sorghum) were ground using a pilot-scale hammer mill (Model D Comminutor, Fitzpatrick Company, Westwood, MA, USA). The initial grain grinding was performed using a 1.65 mm screen to break the grain kernels. This was followed by secondary grinding to achieve different particle sizes using a 1.27 mm screen for coarse grinding, a 1.02 mm screen for medium grinding and a 0.61 screen for fine grinding. The grinding was performed in 200-pound batches to provide replication. The ground grains were then mixed with the other ingredients in a double ribbon mixer (Wenger Manufacturing, Sabetha, KS, USA). Diets were mixed for 3 min for mix uniformity. The mixed diets were post-ground through the same hammermill using the same three screens as described above in order to obtain diets with three different particle sizes for each formulation. The goal of using three different final hammer screen sizes was not to achieve specific or the same particle sizes for the wheat and sorghum-based raw diets. Instead, the screen size was an independent process variable in the experimental design with three levels. Particle size standardization was not targeted, as their distributions are impossible to control precisely. Different ingredients are expected to have varying responses to particle size reduction for the same hammer mill screen size, which was partly what this study investigated. The use of different screen sizes led to distinct particle sizes depending on the type of grain, as described later. Throughout the manuscript, the term screen size is used interchangeably with grind size as an independent variable, and distinguished from actual particle size that was a dependent variable.

### 2.4. Particle Size Analysis

Particle size analysis was done using a rotating-tapping sieve system (Ro-Tap, Tyler Co., Mentor, OH, USA) according to the standard ASAE S319.2 method of determining the fineness of feed materials by sieving [32]. The Ro-Tap test is performed using a series of sieves with decreasing screen size (beginning with a #6 screen and ending with a #270 screen) in the sieve stack. One hundred grams of material was placed on the top sieve and the shaker was turned on and ran for 10 min. The material that remained on top of each sieve post-test was weighed and recorded for the particle size distribution calculations. The geometric mean diameter (dgw) of the samples were calculated based on the particle size distribution.

### 2.5. Rapid Visco Analysis

The pasting properties of the formulations were characterized using a Rapid Visco Analyzer (RVA 4500, Perten Instruments, Waltham, MA, USA), which recorded viscosity changes in slurry form during a controlled heating and cooling process with the application of moderate shear. Testing methodology was based on the method described in a previous study [33]. Briefly, ~3 g of each sample was combined with ~25 mL deionized water to create a 14% (*w*/*v* basis) suspension and placed in the RVA. Stirring was carried out throughout the test as per the standard. The temperature cycle involved holding at 50 °C for 1 min, heating to 95 °C at 12.2 °C per min, followed by holding again at 95 °C for 2.5 min, and cooling to 50 °C at 11.8 °C per min before a final holding period of 2 min. Parameters such as pasting temperature and peak viscosity were recorded using the pasting profiles that were generated from each test. RVA analyses were conducted in duplicate for each grain after secondary grinding through the 3 screens, and also for each mixed diet after post-mix grinding through the 3 screens.

### 2.6. Extrusion

**Figure 1:**
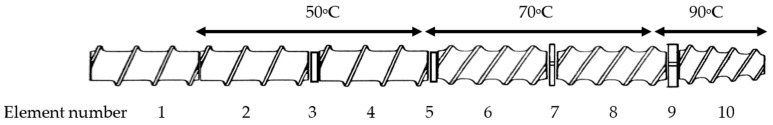
Pilot-scale single screw extruder screw profile and set barrel temperatures, modified from [23].

All diets were processed using a pilot-scale single screw extruder (X-20, Wenger Manufacturing, Sabetha, KS, USA) powered by a 37.3 kW (50 hp) motor. Raw material mash in the live-bottom feed hopper was maintained at medium fill level and the feeder screw speed was set at 10 rpm, which delivered dry mash to the preconditioning system at a rate of 76.25–86.6 kg/h based on pre-extrusion calibration of the volumetric feeding system. Water and steam were injected into the differential diameter and speed pre-conditioner (Wenger model 2 DDC) at rates of 10.34–11.91 kg/h and 12.32–12.85 kg/h, respectively. The extruder barrel, with a L/D ratio of 10:1 and screw diameter of 82.6 mm (3.25 in), was segmented into three heating zones (set at 50, 70 and 90 °C from inlet to discharge) spread over 6 heads. The inlet head had a smooth internal barrel liner, while the rest of the heads had spiral liners. The screw profile (Figure 1) was designed to have an efficient intake of pre-conditioned feed followed by a progressive increase in compression as the melt was conveyed towards extruder discharge, using elements ranging from single-flighted full pitch screws to double-flighted half-pitch screws and a conical screw. Two steam locks were used in the latter half of the screw profile, interspersed between regular screw elements, to increase resistance to flow and barrel fill, and thus provide adequate mechanical energy.

The extruder screw speed was kept constant at 414 rpm. Water was injected at the inlet end of the extruder barrel at a rate of 9.1–12.3 kg/h, to achieve an in-barrel moisture of 36–37% wb. A thermocouple inserted into the material stream immediately upstream of the extruder die recorded the die temperature. A single die insert with nine circular cross-section openings, each with 2 mm exit diameter, was used at the end of the extruder. Extruded products were cut using a rotary knife system with 6 hard blades at a speed of 2141 rpm. The extruded pellets were transferred pneumatically to a pilot-scale two-pass gas-fired continuous dryer and single-pass cooling system (Wenger series 4800). Dryer air temperature was set at 104.4 °C (220 °F) and residence time at 16 min, while the cooler used room temperature air with a product residence time of 5 min.

Specific thermal energy (STE) input during preconditioning was calculated from net thermal energy or heat transferred to the material as follows.

(1) $$STE\;\left(\frac{kJ}{kg}\right)=\frac{Q_{thermal}}{m_{f}}$$

where *m_f_* is the dry feed rate or rate of material delivered by the feeder screw in kg/s, as determined from feed rate calibration; and *Q_thermal_* is the net rate of thermal energy transferred (kJ/s), which was calculated from the difference between the heats of steam injected and unabsorbed steam lost to the atmosphere at the preconditioner discharge, as described previously [23].

Specific mechanical energy (SME) during the extrusion process was calculated as described below [34].

(2) $$SME\;\left(\frac{kJ}{kg}\right)=\frac{W-W_{0}}{m_{f}}$$

where *W* is the power consumed by the extruder motor during processing (kW) and *W*_0_ is the power consumed at no load with no material flowing through (kW), both measured using a wattmeter.

### 2.7. Pellet Expansion Characteristics

Extruded product bulk density was measured by recording the bulk mass of pellets (g) filling a 1 L cup to the brim. Three replicate measurements were made using product directly from the extruder discharge.

(3) $$Bulk\;density\;(\frac{g}{L})=\frac{bulk\;mass\;of\;pellets}{bulk\;volume\;(1\;L)}$$

For piece-wise characterization of expansion, the section expansion index (SEI) and piece density were determined using 20 representative pellets from each treatment. The dimensions of the pellets (cm) were measured with digital calipers, and their mass (g) was also recorded. The SEI of individual pellets was calculated from the pellet diameter $\left(d_{p}\right)$ and die diameter ${(d}_{die})$ as follows.

(4) $$SEI=\frac{d_{p}^{2}}{d_{die}^{2}}$$

The piece density of the pellets was calculated from the pellet mass ${(m}_{p})$ and volume, as described below.

(5) $$Piece\;density\;(\frac{g}{{cm}^{3}})=\frac{m_{p}}{\pi \frac{d_{p}^{2}}{4}l_{p}}$$

where the term in the denominator is the pellet volume assuming a cylindrical shape, and $\left(l_{p}\right)$ is the pellet length.

### 2.8. Pellet Durability

Pellet durability index (PDI) of the dried pellets was determined using the Holman tumble box method as described previously [19]. Screened pellets (500 g) were placed in a metal container with dimensions of 30 cm × 30 cm × 12 cm. The box contained a 23 cm long × 5 cm wide baffle that was centered diagonally inside. Three 1/2” hexagonal metal nuts were added to the box for higher impact on the pellets. The box was rotated at 50 rpm for 10 min, and then the pellets were removed, screened and weighed. The PDI was calculated as follows:

(6) $$\;\;\;PDI\;(\%)=\frac{Weight\;of\;pellets\;after\;tumbling}{Weight\;of\;pellets\;before\;tumbling}\times 100$$

### 2.9. Water Absorption

Water absorption of the dried pellets was measured by selecting 10 pellets randomly, then weighing them and soaking them in 100 mL of room temperature water in a glass beaker for 20 min. At the end of the soaking time, the water was gently decanted, and excess water was blotted off the surface of the pellets using a paper towel. The wet sample was then weighed. Water absorption was calculated from the difference between the two weights as described below.

(7) $$Water\;absorption\left(\%\right)=\frac{Post\;soak\;weight-Pre\;soak\;weight}{Pre\;soak\;weight}\times 100$$

### 2.10. Water Stability

Water stability of the dried pellets was measured using a modified version of a previously reported method [16]. Five grams of pellets were placed in a glass beaker and 100 mL of distilled water at room temperature was added. After one hour, the water was gently decanted from the beaker, and the soaked pellets were dried in a convection oven for 24 h at 90 °C. Water stability was calculated from the weight of the dried pellets as described below.

(8) $$Water\;stability\;(\%)=\frac{Post\;soak\;weight}{Pre\;soak\;weight}\times 100$$

### 2.11. Floating Percentage

Fifty pellets from each treatment were added to a container full of water at room temperature. After 1 min, the pellets that sank to the bottom were counted, and that number was subtracted from the initial count to calculate the floating percentage.

### 2.12. Growth and Digestibility

A 12-week growth trial and a 3-day digestibility study was conducted with Nile tilapia to compare the nutritional efficacy of wheat and sorghum-based practical diets, each prepared by grinding through 3 different hammer mill screen sizes followed by extrusion. Extruded and dried pellets were top-coated with 2% fish oil (based on the final, coated weight) prior to feeding. Proximate compositional analysis for all experimental diets was conducted at MidWest Laboratories, Inc. (Omaha, NE, USA) and presented in Table 2 and Table 3.

The feeding studies were conducted at the E.W. Shell Fisheries Center at Auburn University (Auburn, AL, USA) in an indoor recirculation system. The animal study protocol was approved by the Institutional Review Board of Auburn University (protocol code IACUC 2022-5121). For the growth trial, twenty-four 57 L rectangular glass tanks connected to a common reservoir tank (800 L) were used, with each tank containing twenty juvenile randomly stocked Nile tilapia (mean initial weight = 3.83 ± 0.03 g) that were batch-sorted (group weighed) to uniform size. Four replicate groups per dietary treatment were randomly assigned and diets offered to fish divided into two equal feedings by weight per day. Fish were counted and weighed every other week to adjust the daily feed ration, which was calculated based on expected growth, body weight and feed response. At the end of the 12-week growth trial, fish were counted, and the group was weighed by replicate tank to determine mean final biomass, final weight, survival, percent weight gain, and feed conversion ratio (FCR). Fish were euthanized with 250 mg L^−1^ neutral-buffered tricaine methane sulfonate (MS-222, Syndel, Ferndale, WA, USA) following the recommended dosing protocols. The fish were packed in sealed bags and stored in a freezer (−20 °C) for proximate analysis.

In the digestibility study, apparent digestibility coefficients for dry matter, protein, energy and amino acids were determined by using titanium oxide as an inert marker (1% of the coated weight). The digestibility coefficients of the test diets were determined using groups of 20 fish weighing approximately 81 g each. Fish were allowed to acclimate to the test diets for three days before starting the collection of feces. Fish were offered two feedings and all feces collected using a settling system. Prior to each feeding, the tanks and fecal settling chambers were cleaned. Samples were collected for three days or until a suitable quantity was obtained for analyses (minimum of 1 g dry weight). Daily samples were pooled by tank and four replicate aquaria (n = 4) were utilized for each treatment. Feces were stored in sealed plastic containers and stored in a freezer. Moisture (AOAC 930.15), dry matter, crude protein (AOAC 990.03) [30] and amino acids were determined for the fecal and diet samples according to established procedures. Total energy content of both types of samples was calculated using a micro-calorimetric adiabatic calorimeter bomb using benzoic acid as standard (Model 1425, Parr Instrument Co., Moline, IL, USA). Titanium oxide content analysis followed procedures described previously [35]. Apparent digestibility coefficients of the dry matter, protein and energy for each diet were calculated according to the following formulas [36]:

(9) $$ADMD\;\left(\%\right)=100\;\times [1-\;\frac{\%\;{TiO}_{2}\;in\;feed}{\%\;{TiO}_{2}\;in\;feces}]$$

(10) $$APD\;or\;AED\left(\%\right)=100\times [1-\frac{\%{TiO}_{2}\;in\;feed}{\%{TiO}_{2}\;in\;feces}\times \frac{\%\;nutrient\;in\;feces}{\%\;nutrient\;in\;feed}]$$

where ADMD is apparent dry matter digestibility, APD is apparent protein digestibility, AED is apparent energy digestibility, and TiO_2_ is titanium oxide.

### 2.13. Water Analysis

Dissolved oxygen (DO) was maintained near saturation using air stones in each culture tank via a common airline connected to a regenerative blower. Dissolved oxygen, salinity and water temperature were measured twice daily using a YSI-2030 Pro digital oxygen/temperature meter (YSI Corporation, Yellow Springs, OH, USA), and total ammonia N (TAN) and nitrite-N were measured twice per week using a YSI 9300 photometer (YSI, Yellow Springs, OH, USA). The pH of the water was measured two times per week during the experimental period using a pHTestr30 (Oakton Instrument, Vernon Hills, IL, USA). During the growth trial, DO, temperature, salinity, pH, total ammonia nitrogen (TAN), and nitrite were maintained within acceptable ranges for freshwater fish culture at 6.42 ± 0.06 mg/L, 27.52 ± 1.21 °C, 3.36 ± 0.06 g/L, 6.33 ± 0.16, 0.91 ± 0.22 mg/L, and 0.41 ± 0.24 mg/L, respectively.

### 2.14. Statistical Analysis

Data were analyzed with SAS version 9.4 software (SAS Institute, Cary, NC, USA) using a two-way ANOVA model for determining the statistical significance of treatment and interaction effects (α = 0.05). Following a significant ANOVA result, post hoc comparisons were performed using Tukey’s multiple comparison test to identify significant differences between treatment means (*p* < 0.05).

## 3. Results and Discussion

### 3.1. Grinding and Particle Size

Particle size distribution and average particle sizes of ground grain were clearly impacted by grain type and hammer mill screen size (Table 4). As mentioned earlier, the grains were subject to a two-step size reduction process, first through the 1.65 mm screen and followed by secondary grinding through 0.61, 1.02 or 1.27 mm screens. Expectedly, the average particle size of the ground grain increased with the use of a larger hammer mill screen opening. After the first grinding, the wheat had only a slightly lower average particle size (380 microns) as compared to sorghum (390 microns). After secondary grinding through the lower size screens, the differences between the two grains were further enhanced with markedly lower average particle sizes for wheat than sorghum (196–289 versus 240–348 microns, respectively). The softer or brittle sorghum endosperm led to less energy transfer during milling and shattering of the grain into coarser particles, as opposed to the greater impact and better particle size reduction in wheat that has a harder endosperm due to its gluten proteins [37]. These trends in particle size were an important determinant for several downstream results.

It was apparent that differences arising from grinding of the grains alone had a big role in impacting the particle size distributions of complete wheat- and sorghum-based diets after post-mix grinding through 0.61, 1.02 or 1.27 mm screens (Table 5). The wheat-based diets had lower average particle sizes (203–334 microns) relative to sorghum diets (270–335 microns), although the differences based on grain type were dampened due to the presence of other common ingredients such as soybean meal, corn protein concentrate and fishmeal. The average particle size of both wheat and sorghum diets increased with the use of a larger screen opening, with this trend being more pronounced in the case of wheat.

The particle size distribution became narrower or more uniform with the decrease in screen size for both sorghum and wheat-based mixed diets. A uniform particle size helps ensure a homogenous mix when blending, as large particles tend to self-segregate during agitation [38]. In extrusion, the particle size distribution can also impact process stability, as large particles can plug the die holes, especially when not well hydrated, leading to increased pressure and decreased throughput, and in some cases, complete shutdown, resulting in lost time and increased costs. This is even more critical in aquatic feed production due to the small pellet sizes, and thus the requirement for small die openings. A practical ‘rule of thumb’ is for all particles to be sized below one-third of the die diameter (2.0 mm in the present study). This criterion was met for both wheat and sorghum diets, at least in the case of fine and medium grinds, and was probably not an issue for the coarse grind either, based on the uniform die pressure observed during extrusion as described later.

### 3.2. Rapid Visco Analysis

**Figure 2:**
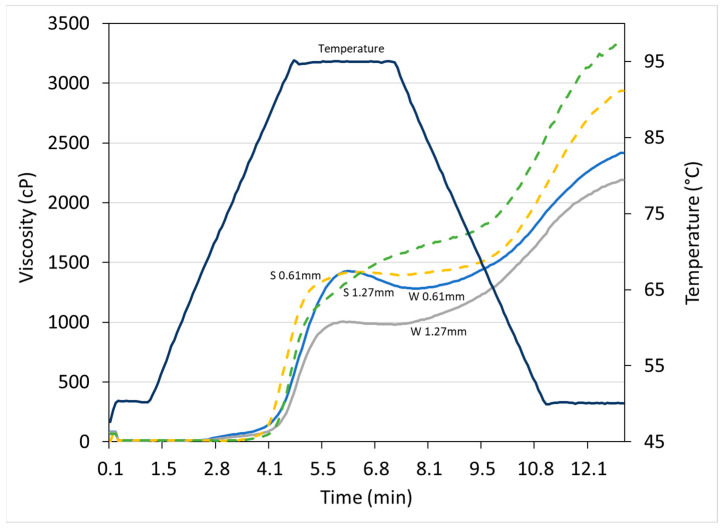
RVA pasting profiles for wheat (W; solid lines) and sorghum (S; dotted lines) ground through 0.61 mm (fine) and 1.27 mm (coarse) hammer mill screens.

Representative RVA pasting profiles of ground grain are shown in Figure 2, which indicate the impact of particle size on the transformation of starch in response to heat, moisture and shear. As the RVA tests progress, starch granules present in the grain absorb water, and as heat is applied, they swell rapidly [39]. Higher pasting temperatures indicate delayed initiation of starch hydration and swelling, and potentially more resistance to gelatinization and degradation [19,23,40]. Ground sorghum grain pasting temperatures for fine, medium and coarse grind were 76.6, 79.8 and 80.7 °C, respectively, and for wheat were 67.8, 68.5 and 69.4 °C, respectively. These ranges were similar to the pasting temperature reported by previous studies for sorghum (78–79 °C) [19] and wheat (71.3 °C) [33]. The higher pasting temperatures for sorghum might be due to the lower accessibility of starch granules bound in the protein matrix, and also its higher particle size that led to delayed hydration, while the lower and/or delayed peak viscosity for wheat, as can be seen from the pasting profiles, is a result of interference in starch–starch interactions by its relatively higher molecular weight proteins. These two conflicting factors are discussed in more detail later. It was also clear that as grind size decreased, the pasting temperatures also reduced for both grains, but the effect was more pronounced for sorghum, as the finer grinding was able to overcome the inhibitory effects mentioned above and allow water to penetrate the starch granules more easily due to the increased surface area of the particles [19]. The trends for mixed diets based on sorghum and wheat were very similar to those for individual grains described above.

### 3.3. Extrusion Processing

Critical extrusion processing parameters are shown in Table 6. As particle size increased, STE increased for both wheat and sorghum-based diets (from 218.6 to 275.5 kJ/kg and from 252.8 to 292.6 kJ/kg, respectively); while on the other hand, SME decreased for both (from 351 to 290.5 kJ/kg and from 358.8 to 267.6 kJ/kg, respectively). The STE trends are contradictory to the expectation of an increase in average surface area with lower particle size and thus a higher absorption of steam. The reduced particle size possibly led to greater water absorption, adhesiveness and material agglomeration in the preconditioner, and actually a higher effective surface area, causing lower steam absorption and STE. Similar results were reported previously in the case of preconditioning process for extruded cat food, with decreasing particle size leading to an increase in steam loss and reduction in absorption [19]. Differences in STE did not impact the preconditioner discharge temperature, which was in the maximum possible range (98–99 °C) across all treatments. Specific mechanical energy during extrusion is an important determinant of product expansion. It impacts both the die temperature and degree of starch transformation, with the former affecting the vapor pressure and the amount of water vapor released, while the latter controls the extensibility of the extruded matrix. Coarser grind sizes led to lower SME because the reduced surface area of the particles inhibited water absorption and energy transfer in the extruder barrel, lowering and/or delaying transformations in the melt, which in turn reduced its effective viscosity and hence the SME. Similar results and reasoning were described in an earlier study focused on the use of corn in pet food [41]. Die temperature is directly impacted by the amount of mechanical energy imparted to the melt, besides some other factors, and thus followed the same trend as the SME, decreasing from 129 to 120 °C for wheat and 124 to 119 °C for sorghum-based diets, as particle size increased.

The sorghum-based diets had overall higher STE than wheat. This can be attributed to the overall larger particle size of the former. For example, after grinding through the finest screen (0.61 mm), the sorghum-based diet had an average particle size of 270 microns in comparison to the 203 microns for the wheat diet. This led to lower agglomeration of the former during preconditioning and better steam absorption, as reasoned above. Except for coarsely ground diets, sorghum-based diets led to marginally more mechanical energy during extrusion than wheat-based diets. This was opposite to the particle size trend discussed earlier, indicating that differences in grain chemistry and morphology might also have an important role. These differences are discussed in more detail in the next section, but are related to the high molecular weight wheat proteins potentially causing relatively more interference in melt viscosity development at finer grinds, and the inaccessibility of sorghum starch at the coarsest grind size. It should also be noted that the die pressure remained constant across all treatments at 250 psi, indicating that irrespective of differences in melt development and rheology across the extruder barrel length, the final viscosity at the die was comparable.

### 3.4. Pellet Expansion Characteristics

Pellet diameter ranged from 2.41 to 2.86 mm and length from 4.04 to 6.28 mm. Pellet expansion data are presented in Table 7. Sorghum-based pellets were less expanded compared to wheat for the medium and coarse grinds, as can be seen from the higher bulk density of the former. Sectional expansion index and piece density trends were also similar. The SEI is a useful way to quantify the degree of expansion in the radial direction of individual pellets. Additionally, piece density is a direct measure of volumetric expansion of individual pellets compared to bulk density, that is a bulk measurement based on packing of hundreds of pellets in a cup. In the sorghum grain endosperm, starch granules are typically tightly bound in the protein matrix [24,42]. Thus, the starch is relatively inaccessible to hydration and gelatinization in sorghum, as compared to the starch in wheat endosperm. This effect was further enhanced by the higher particle size of ground sorghum used in the diets, hence explaining the difference in expansion.

In the case of sorghum-based diets, as the particle size decreased, SEI went up (from 1.46 to 2.15) and piece density and bulk density decreased (from 0.59 to 0.44 g/cm^3^ and from 452 to 367 g/L, respectively), indicating increased radial and volumetric expansion. It is likely that as particle size decreased, the starch in the endosperm was made more accessible and the increase in surface area also led to enhanced hydration and heat penetration, thus greater gelatinization and accompanying transformations and higher pellet expansion upon exiting the die. The increase in SME and die temperature with the decrease in grind size, as discussed in the previous section, also exacerbated this effect, even though the STE decreased, indicating that the former had a greater role in determining expansion. For wheat, however, no particular trends were found in the SEI (ranging from 1.85 to 2.06), piece density (ranging from 0.44 to 0.52 g/cm^3^) and bulk density (ranging from 423 to 444 g/L), despite the differences in SME and STE across the three grind sizes. This difference in trends between wheat and sorghum with respect to particle size is statistically significant (*p*-value for interactions = 0.0003 to 0.0031). The greater sensitivity to particle size when it comes to the expansion of sorghum-based diets is likely due to how the starch granules are embedded in the endosperm, as discussed above.

It is interesting to note that contrary to trends for medium and coarse grinds, sorghum-based pellets expanded more than wheat at the smallest grind. It is likely that the detrimental impact of higher-molecular-weight wheat proteins became a more dominant factor, once the fine-grinding negated the sorghum starch inaccessibility effect.

### 3.5. Pellet Quality Parameters

Pellet durability index is an important indicator of feed quality, as the generation of fines is a significant problem in the industry due to the widespread use of pneumatic conveying [43], which in turn contributes to poor water quality, increased costs and labor at the farm, and decreased health and potential mortality issues for the fish [44]. Feed handling in aquaculture differs from traditional animal feeding. Traditionally, fines were generated through abrasion in the bags during handling. In modern aquaculture, most feed is pneumatically conveyed to the fish, which in fact can generate more fines through substantial impact force [43]. The extruded aquatic feed pellets in this study were extremely durable, with a PDI greater than 98% across all experimental treatments. No significant impact (*p* = 0.1570) or even trends were observed for PDI with respect to particle size for both wheat and sorghum-based pellets. PDI is directly related to the extent of binding of the various components in the feed. In the absence of any artificial binders, gelatinized starch serves as the primary binding agent. It was obvious that the extrusion process sufficiently gelatinized starch, resulting in high pellet durability, irrespective of the particle sizes. Grain type, however, did impact particle size significantly (*p* = 0.0104), although to a marginal degree. Wheat-based pellets had only a slightly lower PDI (pooled average 98.39%), as compared to sorghum-based pellets (98.80%). This difference might be due to the overall higher expansion of the wheat-based pellets, rendering them microstructurally weaker, although this was compensated to some extent by the relatively higher degree of starch transformation relative to sorghum. The tumbler box method used in this study might not be vigorous enough compared to other methods, such as the Holmen air test or the DORIS test (Durability on a Realistic Test) that have led to bigger contrasts between treatments [44,45], but the latter were not used due to reasons including the small pellet size.

The water absorption of extruded pellets, which ranged from 210 to 358, is an important aquatic feed pellet quality metric, which can relate to potential nutrient loss through leeching and increased pellet size, causing feeding issues. It should be noted that the top coating of 2% of fish oil added prior to feeding was absent when these measurements were conducted. With the coating, it is likely that water absorption would decrease, as oil is hydrophobic. It can be seen from pooled data in Table 8 that sorghum-based pellets had significantly lower water absorption (256%) than wheat-based pellets (334%). In low or moderate shear processing situations, wheat is known to absorb more water due to its gluten proteins as compared to sorghum [46,47]. However, this is not the case under the more aggressive high shear and temperature conditions during extrusion, due to protein denaturation. The water absorption differences between sorghum and wheat-based aquatic feed are better explained by the overall lower expansion and porosity of the former. Another factor was the relative inaccessibility of sorghum starch that is bound in the protein matrix as discussed earlier, which might have allowed less water to penetrate. Water absorption significantly increased from 258% to 329% as the particle size decreased. This trend can also be related to the higher expansion or porosity of the pellets and greater exposure of starch during processing with reduced particle size of the raw material. These effects were more prominent for sorghum (increase in water absorption from 210 to 301%) as compared to wheat (307 to 358%), although the interaction between grain type and grind size was not found to be significant (*p* = 0.1877).

All aquatic feed pellets remained physically intact to an acceptable degree after soaking in water for an hour, as inferred from the water stability range of 79–82% across the six treatments. As noted earlier, the top coating by 2% fish oil (added only later for the feeding trials) would likely have further increased water stability. As in the case of pellet durability index, starch has a critical role in determining water stability with an even higher degree of transformation required to counter hydrolysis. A partial breakdown of starch granules beyond just gelatinization ensures improved inter-molecular interactions and enhanced binding in the presence of water. Neither grain type nor grind size had a statistically significant impact on water stability, although some trends were observed. Water stability of sorghum-based pellets was only a little lower (79–82%) than wheat (82%). However, the former slightly increased as the particle size of raw diets decreased, while no differences were observed in wheat-based pellets. These results were explained by the lesser extent of starch transformations in sorghum during processing due to the higher particle size and also the relative inaccessibility of its starch granules [24,42]. Reducing the grind size countered this effect and aided in the release of starch from the protein matrix.

All feeds ranged from 98 to 100% floating, with no trends observed with respect to grain type or grind size, indicating that product density was adequate across all treatments to obtain almost full buoyancy. Tilapia can consume both floating and sinking feed, but growers prefer floating feed to monitor feed intake and also minimize deposition of unconsumed feed at the bottom of the tanks.

### 3.6. Growth and Digestiblity

No disease occurrences were observed, and water quality parameters were within suitable ranges during the growth and digestibility trials for Nile tilapia that were fed the extruded feed. Digestibility results are shown in Table 9. The apparent energy digestibility coefficient ranged from 91.52 to 92.86%, which was much higher than the AED reported for Pacific white shrimp (74.29–82.93) in a previous study by the authors [24]. Physiological and metabolic differences between species is an important factor; however, the high energy intensity in extrusion processing does improve nutrient digestibility and performance in animals [48]. In the case of tilapia feed, the processing energy input is higher than shrimp feed due to the requirement for floating pellets for the former, which could be another factor. The two other digestibility parameters for tilapia measured in this study, APD and ADMD, ranged from 84.09 to 87.15% and 77.92 to 81.11%, respectively.

There was limited evidence of the impact of grain type and grind size on the various digestibility coefficients, but not in a statistically consistent manner. Sorghum-based diets had a significantly lower AED than wheat (pooled average of 85.13% versus 86.42%), but APD and ADMD did not show any statistical differences with respect to grain type. Previous research regarding sorghum as an aquatic feed ingredient is very limited, although there have been extensive studies on its use in traditional livestock. Sorghum was reported to have lower digestible protein (sorghum ~7% versus wheat ~10%), but higher total digestible nutrients (86% versus 78%) and more metabolizable energy (3093 versus 2820 kcal/kg) [49]. However, many factors such as growing conditions, variety, and processing, and also target animal species, influence the nutritional quality, and these data are not universally applicable. The shrimp study mentioned before compared sorghum and wheat-based diets, and also reported lower apparent energy digestibility for the former (76.79 versus 82.93) due to the reduced accessibility of starch bound in the protein matrix of the sorghum endosperm [24]. On the other hand, grind size did not affect AED or APD, but was found to have a significant impact on apparent dry matter digestibility, with medium grind showing the highest ADMD (pooled average of 80.34% versus 78.93 and 78.23% for fine and coarse grinds, respectively). This is partly aligned with previous research showing an increase in the digestibility of sorghum-based dog food with a decrease in particle size due to greater surface area and endosperm exposure during processing [29]. However, contrary to this key research hypothesis, the lowest grind did not show the best digestibility, which could be due to the highest water absorption and the potential leaching of micronutrients leading to negative effects on overall nutrient utilization efficiency and digestibility. It should also be noted that the feed retention time in the gut was not measured in the study; typically, total gastrointestinal transit time for Nile tilapia can be as high as 20 h, with significant emptying of the stomach occurring within the first 4 h [50].

Aquatic species are very dependent upon protein for growth and normal physiological function. It is interesting to note that neither grain type nor grind size had a significant impact on apparent protein digestibility. This could be based on a combination of reasons, including diets not being dependent upon grain for dietary protein due to the inclusion of other sources such as soybean meal, fish meal and corn protein concentrate, and the masking of other effects due to the improvement in protein digestibility by extrusion, as shown in other livestock industries. There are 10 nutritionally essential amino acids (AAs) reported for tilapia: arginine, histidine, isoleucine, lysine, methionine, phenylalanine, threonine, tryptophan and valine [51]. The limiting AA in sorghum is lysine [52]. Lysine is present in fishmeal and in soybean meal, although the latter can be deficient in methionine [53]. Apparent amino acid digestibility coefficients were also similar across all treatments, including for the essential AAs, regardless of grain source or particle size, as shown in Table 10.

Results from the growth trial are given in Table 11. Extremely high survival rates (99–100%) were observed for tilapia during the 12-week feeding period with no significant differences between treatments. Grain type had a marked impact on final overall biomass, final weight, weight gain and % weight gain of tilapia, with sorghum-based extruded diets showing a statistically significant improvement over wheat across all these parameters. Starting from a mean initial animal weight of 3.83 g, the final weights in the case of sorghum-based diets ranged from 81.94 to 88.93 g as compared to 74.42 to 80.63 g for wheat, which corresponded to % weight gains of 2218–2031 and 1850–1985, respectively. This result was contrary to one of the hypotheses for this research. However, it could be explained on the basis of feed intake per fish, which was significantly higher for sorghum-based diets (84.51–87.00 g) than wheat (79.36–85.31 g). This led to a lack of a significant difference between wheat and sorghum-based diets for feed conversion ratio (overall range of 1.03–1.13), which is more in alignment with the digestibility trends discussed earlier. The interesting differences in feed intake data were probably a result of the greater expansion and size of wheat-based pellets, which increased further after swelling due to much higher water absorption. This, in turn, might have led to problems in feeding. So, in essence, even with FCR being statistically similar between sorghum and wheat-based extruded aquatic feed, the feeding performance of the former can be considered to be superior, as it led to greater weight gain in tilapia.

Grind size did not have a significant impact on final overall biomass, weight gain, feed intake per fish or FCR. However, the medium grind treatments showed better performance in all these metrics. This is consistent with the digestibility trends, and as discussed in that context, it can be attributed to the balance between factors relating to endosperm exposure, pellet size and swelling due to water absorption as a function of particle size reduction.

Proximate analysis data representing the whole-body composition of tilapia are shown in Table 12. Tilapia moisture content ranged from 66.75 to 69.9%, crude protein from 15.03 to 16.3%, fat from 11.2 to 12.48% and ash from 1.83 to 3.98%. Grain type and grind size did not lead to statistical differences in body composition, except in the case of ash content, which was significantly impacted by the former. Previous research has shown that dietary protein levels can affect the body composition of tilapia [54]; however, all diets in this study were formulated with 36% crude protein, and whole-body composition is in line with expectations.

## 4. Conclusions

Sorghum inclusion at approximately 30% level in extruded tilapia feed did not result in any negative impact on the physical quality of pellets, and in fact led to lower water absorption and increased pellet durability as compared to wheat. The inclusion of sorghum did not negatively impact growth rates in juvenile tilapia. While the feed conversion ratio was not significantly different, the final overall biomass and weight gain per fish were significantly higher for sorghum-based diets. Apparent protein digestibility was not impacted by the inclusion of sorghum, but wheat-based diets showed slightly higher apparent digestibility of energy. However, growth rates and digestibility are not always linked, as shown by data in this study. Tilapia fed the sorghum-based diets grew significantly more than wheat-based diets, primarily due to increased feed intake. This positive impact on growth rates in tilapia make sorghum a viable and promising alternative to wheat in tilapia feed. Finally, particle size reduction was shown to have a significant impact on the physical quality of the feed, but excessive grinding did not improve fish growth rates, feed conversion ration or digestibility, offering producers the opportunity to save time and costs through improved manufacturing practices by reducing grinding intensity.

## Figures and Tables

**Table 1:** Dietary formulations for extruded tilapia feed. Percentages of soybean meal, soy oil, and ground grain differed slightly between the two diets in order to achieve iso-protein and iso-fat diets.

Ingredients	Wheat-Based Diet	Sorghum-Based Diet
Menhaden Fishmeal	6	6
Soybean Meal	44	44.6
Corn Protein Concentrate	9	9
Menhaden Fish Oil	2	2
Soy Oil	3.52	3
Soy Lecithin	1	1
Ground Wheat/Sorghum	32.32	32.24
Mineral Premix	0.07	0.07
Vitamin Premix	0.04	0.04
Choline Chloride	0.2	0.2
Rovimix Stay-C 35%	0.1	0.1
Dicalcium Phosphate	1.75	1.75
Total	100	100

**Table 2:** Proximate and mineral composition of practical tilapia diets based on sorghum (S) and wheat (W) ground through different hammer mill screen sizes (0.61, 1.02 and 1.27 mm). All data are means from duplicates.

Proximate Composition (g/100 g as Is)	S 0.61	S 1.02	S 1.27	W 0.61	W 1.02	W 1.27
Protein (crude) %	37.7	38.25	38.35	37.65	37.9	38.35
Moisture %	5.55	5.425	6.115	4.33	4.78	5.04
Fat %	7.4	7.57	7.465	5.175	7.8	7.395
Ash %	7.23	7.225	7.25	6.94	7.365	7.285
Sulfur %	0.365	0.37	0.355	0.355	0.37	0.365
Phosphorus %	1.03	1.02	0.97	0.945	1.04	1.04
Potassium %	1.31	1.3	1.24	1.25	1.32	1.275
Magnesium %	0.21	0.21	0.2	0.19	0.2	0.2
Calcium %	0.945	0.93	0.855	0.825	0.89	0.95
Sodium %	0.06	0.07	0.07	0.07	0.07	0.07
Iron %	215	218.5	201	189.5	206.5	211.5
Manganese %	50.5	48.45	46.45	54.55	56.85	55.65
Copper %	41.445	45.55	38.7	43.2	42.85	39.7
Zinc %	70.3	72.25	66.15	73.45	74.65	71.05

**Table 3:** Amino acid composition of practical tilapia diets based on sorghum (S) and wheat (W) ground through different hammer mill screen sizes (0.61, 1.02 and 1.27 mm). All data are means from duplicates.

Proximate Composition (g/100 g as Is)	S 0.61	S 1.02	S 1.27	W 0.61	W 1.02	W 1.27
Alanine	2.09	2.18	2.16	1.91	1.87	1.93
Arginine	2.12	2.14	2.07	2.19	2.14	2.16
Aspartic Acid	3.39	3.5	3.38	3.34	3.34	3.37
Cysteine	0.53	0.58	0.56	0.59	0.57	0.58
Glutamic Acid	7.12	7.44	7.3	7.53	7.5	7.61
Glycine	1.51	1.57	1.58	1.59	1.56	1.58
Histidine	0.87	0.91	0.88	0.88	0.88	0.89
Hydroxylysine	0.02	0.02	0.03	0.03	0.02	0.02
Hydroxyproline	0.11	0.16	0.16	0.11	0.08	0.12
Isoleucine	1.71	1.82	1.76	1.72	1.77	1.71
Lanthionine	0	0	0	0	0	0
Leucine	3.53	3.69	3.6	3.33	3.25	3.36
Lysine	1.87	1.95	1.87	1.91	1.92	1.9
Methionine	0.63	0.65	0.63	0.62	0.61	0.63
Ornithine	0.03	0.03	0.03	0.03	0.03	0.03
Phenylalanine	1.87	1.95	1.9	1.86	1.85	1.88
Proline	2.1	2.21	2.16	2.19	2.14	2.16
Serine	1.55	1.64	1.61	1.53	1.53	1.62
Taurine	0.17	0.16	0.16	0.17	0.17	0.16
Threonine	1.29	1.35	1.31	1.27	1.28	1.32
Tryptophan	0.43	0.42	0.4	0.46	0.46	0.46
Tyrosine	1.35	1.22	1.21	1.38	1.21	1.25
Valine	1.78	1.83	1.77	1.78	1.76	1.74

**Table 4:** Particle size distribution (%) and average particle size (microns) of wheat and sorghum ground through different hammer mill screen sizes. The 1.65 mm screen was used for preliminary grinding followed by secondary grinding using 0.61, 1.02 and 1.27 mm screens.

Particle Size (Microns)	Wheat				Sorghum			
	0.61 mm	1.02 mm	1.27 mm	1.65 mm	0.61 mm	1.02 mm	1.27 mm	1.65 mm
2380	0.00	0.00	0.00	0.00	0.00	0.00	0.00	0.00
1680	0.00	0.00	0.00	0.00	0.00	0.00	0.00	0.00
1191	0.00	0.31	0.00	2.84	0.00	0.10	0.00	1.20
841	0.43	0.60	3.46	16.86	0.30	0.32	3.50	18.00
594	0.53	7.37	18.99	19.17	1.29	12.45	27.01	24.37
420	6.76	22.39	20.32	16.06	15.25	25.62	23.40	15.60
297	23.96	18.12	14.47	11.64	24.70	16.34	11.80	8.60
212	16.93	12.85	10.49	8.40	18.65	11.56	7.38	6.15
150	10.85	8.34	6.40	5.44	19.84	13.74	7.04	5.36
103	26.37	20.06	8.38	7.08	11.79	12.13	12.00	10.75
73	9.13	6.10	10.06	6.97	4.51	4.62	4.14	4.74
53	4.89	3.71	7.15	5.40	3.49	2.98	3.61	4.74
37	0.17	0.18	0.31	0.15	0.17	0.13	0.12	0.46
Average Particle Size	196	255	289	380	240	290	348	390

**Table 5:** Particle size distribution (%) and average particle size (microns) analysis of wheat and sorghum-based tilapia raw diets ground through different hammermill screen sizes.

Particle Size (Microns)	Mixed Diet-Wheat			Mixed Diet-Sorghum		
	0.61 mm	1.02 mm	1.27 mm	0.61 mm	1.02 mm	1.27 mm
2380	0.00	0.00	0.00	0.00	0.00	0.00
1680	0.00	0.00	0.00	0.00	0.00	0.00
1191	0.00	0.00	0.00	0.00	0.00	0.00
841	0.00	0.48	1.73	0.52	0.23	1.92
594	0.35	6.50	21.30	1.52	8.75	22.04
420	7.52	22.40	23.40	10.98	24.70	22.40
297	22.68	18.16	14.74	28.61	18.18	13.30
212	17.26	14.44	11.33	33.69	20.29	11.76
150	18.20	16.46	10.65	16.04	18.02	12.30
103	25.40	16.46	12.97	7.08	8.41	14.49
73	4.75	3.65	3.19	1.21	1.28	1.54
53	3.80	1.28	0.60	0.23	0.07	0.18
37	0.04	0.18	0.08	0.13	0.06	0.08
Average Particle Size	203	266	334	270	303	335

**Table 6:** Critical extrusion processing parameters for tilapia feed manufactured with different grains and grind sizes.

	Grind Size (mm)	STE (kJ/kg)	SME (kJ/kg)	Die Temperature (°C)
Wheat	0.61	218.6	351.0	129
	1.02	254.6	272.3	123
	1.27	275.5	290.5	120
Sorghum	0.61	252.8	358.8	124
	1.02	278.5	285.1	122
	1.27	292.6	267.6	119

Note: STE = specific thermal energy input; SME = specific mechanical energy input.

**Table 7:** Expansion characteristics of extruded aquatic feed pellets manufactured with different grains and grind sizes (mm).

		SEI	Piece Density (g/cm^3^)	Bulk Density (g/L)
Wheat	0.61	1.85 ^bc^	0.52 ^b^	444.0 ^a^
	1.02	2.06 ^ab^	0.44 ^c^	423.0 ^a^
	1.27	1.87 ^bc^	0.47 ^bc^	430.5 ^a^
Sorghum	0.61	2.15 ^a^	0.44 ^c^	367.2 ^b^
	1.02	1.65 ^cd^	0.53 ^ab^	435.0 ^a^
	1.27	1.46 ^d^	0.59 ^ab^	452.5 ^a^
*p*-value	Grain:	0.0035	0.0080	0.0556
	Grind	0.0009	0.0048	0.0067
	Interaction:	0.0003	0.0003	0.0031

Note: SEI = section expansion index. Values within each column with different letters are significantly different (*p* < 0.05) based on two-way ANOVA followed by Tukey’s multiple comparison test.

**Table 8:** Water absorption (%) of extruded aquatic feed pellets manufactured with different grains and grind sizes (mm).

Main Effect	Grain/Grind Size	Water Absorption %
Grain	Wheat	334.4 ^a^
(*p*-value < 0.0001)	Sorghum	255.7 ^b^
Grind Size	0.61	329.3 ^a^
(*p*-value < 0.0001)	1.02	297.6 ^b^
	1.27	258.3 ^c^

Note: Values represent pooled data. Different letters within each factor represent a significant difference (*p* < 0.05).

**Table 9:** Apparent digestibility coefficients (%) for dry matter (ADMD), protein (APD) and energy (AED) for extruded diets manufactured with different grains and grind sizes fed to tilapia.

		ADMD	AED	APD
Wheat	0.61	79.01 ± 0.67	86.10 ± 1.54 ^ab^	92.79 ± 0.57
	1.02	81.11 ± 0.78	87.15 ± 1.00 ^a^	92.86 ± 0.24
	1.27	78.52 ± 0.88	86.00 ± 0.88 ^ab^	92.38 ± 0.67
Sorghum	0.61	78.84 ± 3.31	85.75 ± 0.40 ^ab^	92.12 ± 1.28
	1.02	79.56 ± 0.67	85.53 ± 0.96 ^ab^	92.65 ± 0.45
	1.27	77.92 ± 0.57	84.09 ± 1.03 ^b^	91.52 ± 0.69
*p*-value	Grain:	0.2235	0.0065	0.0676
	Grind	0.035	0.0595	0.1102
	Interaction:	0.6533	0.2907	0.6683

Note: Values from each diet are means and ±SD of four tanks. Values within each column with different letters are significantly different (*p* < 0.05) based on two-way ANOVA followed by Tukey’s multiple comparison test.

**Table 10:** Apparent amino acid (AA) digestibility (%) for extruded sorghum (S)- and wheat (W)-based diets manufactured with different raw material grind sizes (mm) fed to tilapia.

AA	S 0.61	S 1.02	S 1.27	W 0.61	W 1.02	W 1.27
Alanine	92.28 ± 1.63	93.23 ± 0.89	92.47 ± 1.09	93.622 ± 0.55	93.57 ± 0.31	93.46 ± 0.50
Arginine	96.48 ± 0.43	96.36 ± 0.53	96.02 ± 0.29	96.85 ± 0.36	96.73 ± 0.25	96.24 ± 0.31
Aspartic Acid	95.39 ± 0.86	95.67 ± 0.33	95.00 ± 0.38	95.96 ± 0.25	95.80 ± 0.12	95.42 ± 0.34
Cysteine	93.09 ± 1.31	93.92 ± 0.11	93.09 ± 0.60	94.75 ± 0.15	94.37 ± 0.10	94.16 ± 0.46
Glutamic Acid	96.03 ± 0.69	96.41 ± 0.38	95.57 ± 0.54	97.29 ± 0.25	97.14 ± 0.18	96.97 ± 0.30
Glycine	89.14 ± 1.89	89.77 ± 2.43	89.85 ± 1.27	90.91 ± 0.81	91.06 ± 0.68	90.15 ± 0.87
Histidine	95.14 ± 0.81	95.62 ± 0.39	95.16 ± 0.41	96.06 ± 0.40	95.98 ± 0.16	95.72 ± 0.17
Hydroxylysine	62.74 ± 15.16	69.13 ± 9.34	76.03 ± 4.18	82.53 ± 3.98	74.11 ± 4.32	73.06 ± 6.81
Hydroxyproline	59.26 ± 8.78	72.98 ± 9.57	72.70 ± 3.67	66.57 ± 4.37	57.55 ± 4.83	62.82 ± 3.08
Isoleucine	93.42 ± 1.25	94.44 ± 0.25	93.66 ± 0.49	94.68 ± 0.55	94.90 ± 0.24	94.47 ± 0.36
Leucine	93.63 ± 1.26	94.50 ± 0.46	93.66 ± 0.75	94.90 ± 0.51	94.97 ± 0.25	94.93 ± 0.33
Lysine	96.07 ± 0.66	96.35 ± 0.29	96.28 ± 0.19	96.37 ± 0.26	96.26 ± 0.11	95.87 ± 0.16
Methionine	95.19 ± 0.95	95.67 ± 0.48	95.09 ± 0.38	96.01 ± 0.60	95.67 ± 0.11	95.82 ± 0.30
Ornithine	89.72 ± 3.13	93.18 ± 0.22	92.64 ± 0.19	93.00 ± 0.22	90.64 ± 3.33	90.98 ± 3.89
Phenylalanine	93.80 ± 1.11	94.47 ± 0.38	93.72 ± 0.55	94.86 ± 0.49	94.87 ± 0.27	94.74 ± 0.26
Proline	92.53 ± 1.18	93.00 ± 0.97	92.14 ± 0.91	94.31 ± 0.63	94.33 ± 0.24	93.93 ± 0.36
Serine	93.97 ± 1.17	94.91 ± 0.61	94.19 ± 0.72	95.20 ± 0.20	95.07 ± 0.15	94.89 ± 0.36
Taurine	84.01 ± 3.73	82.46 ± 1.30	81.01 ± 1.10	83.68 ± 1.06	85.03 ± 0.94	82.18 ± 1.88
Threonine	89.86 ± 2.17	91.06 ± 0.61	90.26 ± 0.64	90.87 ± 0.42	90.90 ± 0.30	90.81 ± 0.30
Tryptophan	95.61 ± 0.33	95.62 ± 0.14	95.16 ± 0.33	95.99 ± 0.68	95.99 ± 0.26	95.91 ± 0.53
Tyrosine	94.82 ± 0.98	94.13 ± 0.28	93.38 ± 0.48	95.55 ± 0.40	95.00 ± 0.53	94.83 ± 0.44
Valine	92.90 ± 1.51	93.99 ± 0.20	93.16 ± 0.69	94.56 ± 0.66	94.50 ± 0.34	94.29 ± 0.50
Sum AA	94.03 ± 1.09	94.57 ± 0.54	93.88 ± 0.60	95.18 ± 0.40	95.11 ± 0.16	94.82 ± 0.34

Note: Values from each diet are means and ±SD of (n = 4) tanks.

**Table 11:** Response of juvenile Nile tilapia (mean initial weight 3.83 ± 0.03 g) within a 12-week period fed extruded diets manufactured with different grains and grind sizes. Values represent the means of four replicates.

		Final Biomass (g)	Final Weight (g)	Weight Gain (g)	Weight Gain (%)	Total Feed per Fish (g)	FCR	Survival (%)
Wheat	0.61	1469.3 ^b^	74.42 ^b^	70.61 ^b^	1850	79.36	1.13	99
	1.02	1590.9 ^ab^	80.63 ^ab^	76.77 ^ab^	1985	85.31	1.11	99
	1.27	1509.8 ^ab^	75.49 ^ab^	71.75 ^ab^	1918	80.89	1.13	100
Sorghum	0.61	1617.3 ^ab^	81.94 ^ab^	78.10 ^ab^	2031	84.51	1.09	99
	1.02	1778.6 ^a^	88.93 ^a^	85.08 ^a^	2218	86.41	1.03	100
	1.27	1640.5 ^ab^	83.09 ^ab^	79.20 ^ab^	2036	87	1.1	99
*p*-value	Grain:	0.0053	0.0071	0.0071	0.0159	0.0196	0.053	1.000
	Grind:	0.0719	0.1084	0.1078	0.1485	0.1665	0.3406	0.7815
	Interaction:	0.8889	0.9907	0.9878	0.7847	0.4202	0.6465	0.4866

Note: FCR = Feed conversion ratio = feed offered/(final weight–initial weight); values within each column with different letters are significantly different (*p* < 0.05) based on two-way ANOVA followed by Tukey’s multiple comparison test.

**Table 12:** Whole-body composition (on a wet weight basis) of tilapia fed diets containing different extrusion sizes of sorghum and wheat. Values represent the means of four replicates.

		Moisture %	Crude Protein %	Fat %	Ash %
Wheat	0.61	66.75	16.3	11.31	3.98 ^a^
	1.02	67.03	16.13	11.83	3.72 ^a^
	1.27	69.88	15.55	11.2	2.75 ^ab^
Sorghum	0.61	69.3	15.03	12.48	1.83 ^b^
	1.02	69.9	15.83	12.15	2.66 ^ab^
	1.27	68.95	15.9	12.13	3.34 ^ab^
*p*-value	Grain:	0.1779	0.3577	0.313	0.0024
	Grind	0.5667	0.8247	0.9396	0.6603
	Interaction:	0.2989	0.3266	0.9023	0.0022

Note: Values within each column with different letters are significantly different (*p* < 0.05) based on two-way ANOVA followed by Tukey’s multiple comparison test.

## Data Availability

The original contributions presented in this study are included in the article. Further inquiries can be directed to the corresponding author.
